# *Enterococcus faecalis* Isolated From Infant Feces Inhibits Toxigenic *Clostridioides (Clostridium) difficile*

**DOI:** 10.3389/fped.2020.572633

**Published:** 2020-09-25

**Authors:** Chonticha Romyasamit, Anucha Thatrimontrichai, Aratee Aroonkesorn, Wannarat Chanket, Natnicha Ingviya, Phanvasri Saengsuwan, Kamonnut Singkhamanan

**Affiliations:** ^1^Department of Biomedical Sciences, Faculty of Medicine, Prince of Songkla University, Songkhla, Thailand; ^2^Department of Pediatrics, Faculty of Medicine, Prince of Songkla University, Songkhla, Thailand; ^3^Department of Biochemistry, Faculty of Sciences, Prince of Songkla University, Songkhla, Thailand; ^4^Clinical Microbiology, Department of Pathology, Faculty of Medicine, Prince of Songkla University, Songkhla, Thailand

**Keywords:** probiotics, *Enterococcus faecalis*, *Clostridioides (Clostridium) difficile*, spores, intestinal cell

## Abstract

*Clostridioides (Clostridium) difficile* infection is implicated as a major cause of antibiotic-associated diarrhea in hospitals worldwide. Probiotics, especially lactic acid bacteria, are the most frequently used alternative treatment. This study aims to identify potential probiotic enterococci strains that act against *C. difficile* strains and exert a protective effect on colon adenocarcinoma cells (HT-29 cells). To this end, nine *Enterococcus* strains isolated from the feces of breast-fed infants were investigated. They were identified as *E. faecalis* by 16s rRNA sequencing and MALDI-TOF. The probiotic properties including their viabilities in simulated gastrointestinal condition, cell adhesion ability, and their safety were evaluated. All strains exhibited more tolerance toward both pepsin and bile salts and adhered more tightly to HT-29 cells compared with the reference probiotic strain *Lactobacillus plantarum* ATCC 14917. Polymerase chain reaction (PCR) results exhibited that six of nine strains carried at least one virulence determinant gene; however, none exhibited virulence phenotypes or carried transferable antibiotic resistance genes. These strains did not infect *Galleria mellonella* when compared to pathogenic *E. faecalis* strain (*p* < 0.05). Moreover, their antibacterial activities against *C. difficile* were examined using agar well-diffusion, spore production, and germination tests. The six safe strains inhibited spore germination (100 – 98.20% ± 2.17%) and sporulation, particularly in *C. difficile* ATCC 630 treated with *E. faecalis* PK 1302. Furthermore, immunofluorescence assay showed that the cytopathic effects of *C. difficile* of HT-29 cells were reduced by the treatment with the cell-free supernatant of *E. faecalis* strains. These strains prevented rounding of HT-29 cells and preserved the *F*-actin microstructure and tight junctions between adjacent cells, which indicated their ability to reduce the clostridial cytopathic effects. Thus, the study identified six *E. faecalis* isolates that have anti-*C. difficile* activity. These could be promising probiotics with potential applications in the prevention of *C. difficile* colonization and treatment of *C. difficile* infection.

## Introduction

*Clostridioides (Clostridium) difficile* is a Gram-positive rod, spore-forming, anaerobic, and toxin-producing bacterium. *C. difficile* infection (CDI) is a major cause of antibiotic-associated diarrhea (AAD) and hospital-acquired diarrhea, and its manifestations range from mild diarrhea to pseudomembranous colitis and death (1). Globally, the incidence and severity of CDI have substantially increased in the last decade, as indicated by high morbidity and mortality (1, 2).

The spread of *C. difficile* in healthcare settings is usually associated with endospores (3), which are highly resistant to chemicals and extreme temperatures and persist for months and even years. The environment around CDI patients and the large intestine of patients receiving broad-spectrum antibiotics have been found to be contaminated with the spores of *C. difficile*. In the absence of normal flora and under appropriate conditions, the spores mature into vegetative cells in the intestines, which eventually leads to CDI (2). The vegetative cells attach to the epithelial cells and transfer its toxins to the cells. The pathogenesis of CDI is mediated by toxins, such as enterotoxin (TcdA) and cytotoxin (TcdB), which are encoded by *tcdA* and *tcdB* genes, respectively (4). These toxins are major virulence determinants and exhibit both cytopathic and cytotoxic effects on mammalian cells. In intestinal epithelial cells, these effects are mediated by inactivation of the Rho family GTPases, such as Rho, Cdc42, and Rac, leading to disrupted and disorganized F-actin cytoskeleton and tight junctions, morphological changes, and subsequent cell death (4, 5).

Vancomycin and fidaxomicin are usually recommended for CDI. However, recurrence of the infection was reported by up to 24% of patients within 2 months of first episode; the risk of recurrences increased further (50–65%) following subsequent episodes (5, 6). In European and Asian countries, the rate of resistance is more than 60% (7). Consequently, research with alternative prevention or treatment of CDI have gained prominence.

Probiotics are “live micro-organisms which, when administered in adequate amounts, confer a health benefit on the host” (8). Systematic studies have demonstrated that some probiotic bacteria, especially lactic acid bacteria (LAB) and *Bifidobacterium* spp., can affect CDI therapy; probiotics have been shown to prevent AAD (17%) and prevent or treat CDI 3% in a clinical trial (9, 10). Various antimicrobial mechanisms have been attributed to LAB probiotics. These include nutrient competition, prevention of mucosal cell adhesion, and internalization of pathogens such as *C. difficile* (9, 11, 12). Moreover, LAB strains can produce lactic acid and certain antimicrobial molecules such as hydrogen peroxide, fatty acid, and bacteriocins to exert their antimicrobial activity (13, 14). Additionally, bile salt hydrolase (BSH) produced by LAB to transform conjugated bile acid to unconjugated bile acid can inhibit the germination of the spores of *C. difficile* (10, 15).

*Enterococcus* spp. belong to LAB (16) and produce lactic acid and a wide range of bacteriocins (14, 16). The enterococci ubiquitously occur as microflora on the intestinal ecosystem of animals and humans, especially *E. faecalis* and *E. faecium* (17). They are also present in human milk, human vaginal secretions, and fermented foods and dairy products, primarily because of their tolerance to extremes of pH, temperature, and salinity (16). In previous studies, the probiotic strains of *Enterococcus* have been shown to serve as functional foods that mitigate or prevent diarrhea caused by foodborne pathogens such as *Escherichia coli, C. perfringens*, and *C. difficile* (12, 18). Although *E. faecium* NM1015, *E. faecalis* NM815, and *E. faecalis* NM915 have been shown to inhibit *C. difficile in vivo* (12), few studies have examined the anti-*C. difficile* activity of enterococci. In this study, we identified proper enterococci strains that contain not only antibacterial activity against *C. difficile* strains but also probiotic properties. Further, we evaluated their protective effect on the cytopathy of *C. difficile* in HT-29 cells.

## Materials and Methods

### Fecal Sample Collection and Isolation of LAB

Feces samples (*n* = 38) of breast-fed infants in Songklanakarind Hospital were collected with the necessary approval from the Ethics Committees of the Faculty of Medicine, Prince of Songkla University (REC.61-064-4-2). Infants were enrolled according to the following criteria: age < 6 months, exclusively received breast milk with predominant LAB strains, vaginal delivery, healthy infants, mothers without present or past underlying adverse medical conditions, and full-term pregnancy. The feces were immediately cultured on de Man Rogosa Sharpe agar (Merck Millipore, Darmstadt, Germany) at 37°C for 48 h under anaerobic conditions. After incubation, each of the isolated colonies were picked and stored at −80°C in BHI broth with 30% glycerol until testing.

### Bacterial Strains and Culture Condition

Two reference strains, *C. difficile* ATCC 630 (Ribotype 012) and ATCC 43255 (Ribotype 087), obtained from the American Type Culture Collection (ATCC) were used in this study. Two clinical isolates, *C. difficile* 17 and *C. difficile* 541, that were identified using MALDI-TOF MS/MS were obtained from the clinical microbiology laboratory of Songklanagarind Hospital. *C. difficile* strains were cultured on Cycloserine Cefoxitin Fructose Agar (CCFA, Merck Millipore) and the agar plates were incubated at 37°C for 48 h under anaerobic conditions. The colonies were transferred to thioglycolate broth (Merck Millipore) and incubated at 37°C for 18 h. The *C. difficile* strains with different morphologies formed by suspected Enterococcus were stored at −80°C in thioglycolate broth 30% glycerol until testing.

*E. faecalis* DMST 4736 was obtained from the Department of Medical Sciences Thailand (DMST). This strain was cultured on BHI agar and incubated at 37°C for 18 h. *E. faecalis* DMST 4736 were stored at −80°C in BHI broth with 30% glycerol until testing.

### Screening of Fecal Isolates for *C. difficile*

Agar well-diffusion assay was used to test the inhibitory activity of the isolated colonies from feces samples against the toxigenic *C. difficile* according to Nigam et al. (19) with slight modifications. Briefly, overnight cultures of toxigenic *C. difficile* ATCC43255 and ATCC 630 were inoculated on BHI agar and were cut out of the agar. Each well was filled with 50 μl of 1 × 10^8^ CFU/ml of the selected isolates. The plates were incubated at 37°C for 48 h under anaerobic condition and were inspected for the presence of inhibition zones. The tests were performed in duplicate.

### Bacterial Identification

The bacteria were identified using Gram staining, microscopic examination, and catalase activity conducted according to Bergey's manual (20) and confirmed using MALDI-Biotyper® (Karlsruhe, Germany) according to the manufacturer's instructions. Additionally, the isolates were identified by the amplification of their 16S rRNA genes using universal primers 27F and 1492R (21) and sequencing on 6 Applied Biosystems 3730xl (Macrogen, Korea). Sequences were aligned with NCBI database using BLAST search tool to establish sequence similarity (22).

### Characterization of Probiotic Properties

#### Survival Under Gastrointestinal Tract (GIT) Conditions

Tolerance to low pH (pH 2.0, 3.0, and 4.0) and bile salts were tested following the procedure reported by Rodríguez et al. (23). Tolerance to simulated gastric and pancreatic digestion was determined using a reported protocol but with slight modifications (24). Tolerance was determined by mixing 1 ml of stimulated gastric (3 g/L, pH 2) or pancreatic juice (1 g/L, pH 8) with 0.5 ml of BHI broth containing 10^8^ CFU/ml of bacterial cells. The mixtures were incubated at 37°C for 3 h or 4 h for gastric or pancreatic conditions, respectively. The number of colonies on BHI plates before and after incubation with stimulated gastric and pancreatic juices were counted using spared plate method.

$$\begin{array}{l} \text{Survival rate }\left(\%\right)\\ =\;\left[\text{Final }\left(\text{Log CFU}/\text{ml}\right)/\text{Initial }\left(\text{Log CFU}/\text{ml}\right)\right]\;\times \;100 \end{array}$$

#### Cell Surface Hydrophobicity Assay

The hydrophobicity of the isolates was determined using xylene extraction assay (25). The percentage hydrophobicity (H%) was calculated as follows:

$$\begin{array}{l} \text{H\% }=\;\left[\left({\text{A}}_{0}-\text{A}\right)/{\text{A}}_{0}\right]\;\times \;100, \end{array}$$

where A_0_ and A are absorbance values measured before and after xylene extraction.

#### Human Intestinal Cell Adhesion Assay

The adhesion ability of probiotic strains to adhere to the intestinal epithelial cells contributes to their colonization and pathogen exclusion in adhesion to intestinal epithelial cells. The adhesion of bacterial isolates to human colon adenocarcinoma cells (HT-29 cells) was measured as described by Monteagudo-Mera et al. (24). The number of bacteria adhering to the HT-29 cells was calculated as follows:

$$\begin{array}{l} \text{\%Adhesion ability }=\;\left({\text{V}}_{1}\;\times \;100\right)/{\text{V}}_{0}, \end{array}$$

where V_0_ is the initial viable count and V_1_ is the viable count adhered to the HT-29 cells after incubation.

#### Screening for Bacteriocins

Bacteriocins were measured using a modified method (22). Briefly, bacteria (10^8^ CFU/ml) were centrifuged (7,000 × *g* for 10 min), and the pH of the supernatant was adjusted to 6.5 with 1 N NaOH. The neutralized supernatants were incubated with or without 1 mg/ml of proteinase K at 30°C for 2 h and then heated at 80°C for 10 min to inactivate the protease. The supernatants were filtered through 0.2-μm membrane filters. Aliquots of the supernatants were dropped onto the respective BHI agar plates, which were previously covered with an overnight culture of pathogenic indicator bacteria, and incubated aerobically at 37°C for 48 h. Depending on whether or not the test bacteria produced bacteriocins, a small clear zone or no clear zone formed around the wells incubated with 1 mg/ml proteinase K. These were compared with the wells that were not treated with proteinase K.

#### Hydrogen Peroxide (H_2_O_2_) Production and Bile Salt Hydrolase (BSH) Activity

H_2_O_2_ production of the selected isolates (26) and their BSH activities (27) were tested according to reported procedures.

### Safety Assessments

#### Virulence Factors

Genes encoding potential virulence factors were detected using polymerase chain reaction (PCR) amplification methods. The primers are shown in Supplementary Table 1. The phenotypic assays, gelatinase production, hemolytic activity, and mucin degradation were performed as reported earlier (28).

#### Susceptibility to Antibiotics

Antibiotic susceptibility was performed according to the Clinical and Laboratory Standards Institute (CLSI) 2019 guidelines (29). The antibiotics selected for testing include ampicillin (10 μg), penicillin (10 μg), imipenem (10 μg), vancomycin (30 μg), gentamicin (10 μg), erythromycin (15 μg), tetracycline (30 μg), and ciprofloxacin (5 μg).

#### Virulence in the *Galleria mellonella* Model

The *G. mellonella* model was used to determine the toxicity of putative probiotic strains as described previously (30). Briefly, larvae were infected through hemocoel of the last left proleg using a sterilized insulin syringe with 10-μl inocula of different *E. faecalis* strains containing 5 × 10^8^ CFU/ml. *E. faecalis* DMST 4736 as pathogenic strain and PBS were also examined under the same conditions as a virulent control and uninfected control, respectively. After injection, the larvae were incubated in the dark at 37°C for 5 days. The survival of the larvae was recorded every day.

### Evaluation of Potential Probiotic Activity Against *C. difficile* and Its Spore

#### Agar Well-Diffusion Assay

Agar well-diffusion assay was used to test the inhibitory activity of the isolated colonies from feces samples against the toxigenic *C. difficile*, according to reported procedure (19) with slight modifications. Briefly, overnight cultures of toxigenic *C. difficile* strains (*C. difficile* ATCC630, ATCC43255, 17, and 541) were suspended in BHI broth to attain a cell density of 1 × 10^8^ CFU/ml and spread on the BHI agar plates. Five wells (each 9 mm in diameter) were cut out of the agar. Each well was filled with 50 μl of 1 × 10^8^ CFU/ml of a selected isolate. The plates were incubated at 37°C for 48 h under anaerobic conditions, and the inhibition zones were measured. Each test was performed in triplicate.

#### Spore Purification

This method was modified from (31). *C. difficile* was grown on BHI agar overnight at 37°C. A single colony from the BHI agar plate was inoculated in 10 ml of BHI broth with 0.5% yeast extract and 0.1% L-cysteine (Merck Millipore, Darmstadt, Germany) and incubated at 37°C overnight under anaerobic conditions, and 1 ml of the BHI culture was sub-cultured into BHI agar with 0.1% L-cysteine and incubated at 37°C in an anaerobic jar for 7 days. After 7 days of incubation at 37°C, the sporulation efficiency was confirmed by phase-contrast microscopy and measurement of heat-resistant CFU and spore crops harvested immediately or after overnight incubation at 4°C. The spores were washed in PBS two times; suspended in PBS containing 125 mM Tris, 200 mM EDTA, 0.3 mg/ml proteinase K (Amresco, USA), and 1% sarcosyl; and incubated with gentle shaking at 37°C for 2 h. The spores were centrifuged (6,500 × *g*, 10 min) and the pellet was resuspended in water and washed 10 times. After the final suspension in water, the spores were heat-treated (60°C, 20 min) to kill any residual cells. The spore supernatants were stored at 4°C until testing. To calculate the spore CFU, aliquots were serially diluted in PBS and plated onto BHI agar supplemented with 0.1% sodium taurocholate (Merck Millipore). The plates were incubated for 48 h before the enumeration of CFU.

#### Inhibitory Germination Test

The germination test was performed following a method that was modified from reported protocol (32). Briefly, 15 μl of the spore suspension (5 × 10^6^ spores/ml) was added to 96-well plates containing 135 μl of BHI broth and 0.01% taurocholate, with or without 10^8^ CFU/ml specific *E. faecalis* strains and incubated anaerobically at 37°C for 30 min. The germinated spores were enumerated by plating for colony-forming units (CFU) on BHI agar, and percentage germination was calculated as follows:

$$\begin{array}{l} \text{Percentage germination}\\ =\;\left[\text{post-assay CFU}/\text{initial CFU}\right]\;\times \;100. \end{array}$$

#### Sporulation Inhibition Test

Following Carlson et al., inhibition of sporulation was measured (32). Spore formation was evaluated in broth cultures. The log phase cultures of *C. difficile* BHI were inoculated in tryptose yeast extract broth (3% tryptose and 2% yeast extract) at an initial density of 1 × 10^6^ CFU/ml with or without 10^8^ CFU/ml *E. faecalis* strains. After 48 h of culture, the samples were analyzed for the presence of vegetative cells and spores using microscopy. The percentage of sporulation was calculated.

$$\begin{array}{l} \text{\%sporulation}\\ =\;[\text{number of spore}/\left(\text{number of spore }\times \text{ number of vegetative cell}\right]\\ \times \;100 \end{array}$$

#### Co-culture of Probiotics and Toxigenic *C. difficile* With HT-29 Cells

The method reported by Valdes-Varela et al. was adapted to test the influence of probiotics exposure on the cytopathic effects of *C. difficile* on HT-29 cells. The six *E. faecalis* strains were cultured in BHI broth and incubated at 37°C for 18 h under anaerobic conditions. *E. faecalis* cells were washed twice with PBS and resuspended (10^8^ CFU/ml) in the HT-29 cell cultivation medium supplemented with toxigenic *C. difficile* and then incubated for 1 h under anaerobic conditions and stirring (300 rpm). Next, these supernatants were directly used to test their cytotoxicity on HT-29 monolayers; HT-29 cells were seeded onto 96-well tissue culture plates (5 × 10^4^ cells per well). The plates were incubated at 37°C in 5% CO_2_ until a confluent monolayer formed. Twenty microliters of each supernatant was added to the HT-29 cells. The plates were incubated for 24 h at 37°C in 5% CO_2_. Then, the cells were examined under an inverted microscope for morphological changes. The cytopathic effect was indicated by more than 50% of rounded cells.

#### Immunofluorescence Assay

The procedure was adapted from the method reported earlier (33, 34). Briefly, the HT-29 cells subjected to treatments with different supernatants were analyzed using confocal microscopy. For this, the wells in an 8-well plate were seeded with 300 μl (2 × 10^6^ HT-29 cells/ml) and incubated for 20 h to reach the confluent state. Then, the supernatant was removed, and the wells were filled with the same volume of each supernatant containing different bacterial strains with toxigenic *C. difficile* or DMEM medium (negative control). Incubation was continued for an additional 24 h. Then, the supernatant was removed from each well and the HT-29 cells were fixed with 300 μl of 3.7% formaldehyde for 15 min. The samples were washed three times with PBS for 5 min and permeabilized with PBS containing 0.1% Triton X-100 for 15 min. The non-specific binding sites were blocked by treatment with 1% BSA for 20 min, and the cells were washed once again with PBS. The Phalloidin-Alexa-Fluor-488 probe (Invitrogen, USA) toward F-actin was added in 100 μl of PBS (final concentration 1:40), and the samples were incubated for 1 h at 4°C in darkness. After washing three times with PBS, the nuclei of HT-29 cells were stained with DAPI (Sigma Chemical Co.) at 1:1000 (final dilution in PBS) dilution and incubated for 20 min. Finally, the samples were washed and 50 μl of anti-fade mountants (Invitrogen) was added prior to visualization under a Super-Resolution Laser Scanning Confocal Microscope; SR-LSCM (ZEISS, Germany) using a 63×/1.4 oil objective.

### Statistical Analysis

All assays were performed three times independently. Results are expressed as mean ± standard deviation, and the differences between the groups were evaluated by Student's *t*-test or one-way analysis of variance (ANOVA) using GraphPad Prism 5. A *p*-value of < 0.05 was considered statistically significant. The Kaplan–Meier survival function of Stata software was applied to analyze survival (*p* < 0.05).

## Results

### Isolation and Screening for Bacteria Active Against *C. difficile*

Eighty-five distinct colonies of LAB were collected from the feces of 38 breast-fed infants. Of these, nine isolated strains exhibited antimicrobial activity against *C. difficile* ATCC 43355 and ATCC 630.

### Identification of Active Isolates

Primarily, nine cocci-shaped isolates were Gram-positive, catalase-negative, tolerant to 6.5% NaCl, and produced bile esculin. These phenotypic properties indicated that the isolates were enterococci. Further, 16S rRNA sequences of all the isolates showed >97% homology to *E. faecalis*. Moreover, the sequence alignment of 16S RNA genes of isolates among themselves did not display 100% identity, suggesting distinct strains. MALDI-TOF MS analysis of the isolates showed a match with *E. faecalis* strain CLB21560 with a score between 2.302 and 2.443 suggesting that the identification of species was reliable.

### Characterization of Probiotic Features

The survival under GIT conditions requires tolerance to acidic pH, pepsin, pancreatin, and bile salts. These are significant properties of probiotic strains. The survival data for the nine *E. faecalis* isolates and the control probiotic strain [*Lactobacillus plantarum* ATCC14917 (TISTR 877)] under GIT conditions are summarized in Table 1. The nine *E. faecalis* isolates could survive after exposure to pH 2–4 for 2 h; however, a reduction in the survival percentage (67.25 ± 2.01% to 96.55 ± 1.52%) was observed at pH 2. At the same pH, *L. plantarum* ATCC14917 was totally inhibited. When the *E. faecalis* isolates were implanted in pepsin (pH 2), its survival rate (54.06 ± 1.72% to 36.94 ± 1.92%) was significantly higher than that of *L. plantarum* ATCC14917 (non-viable). *E. faecalis* PK2502 showed the highest survival percentage (54.06 ± 1.72%) followed by *E. faecalis* PK1801 (49.97 ± 1.47%) and *E. faecalis* PK2004 (46.55 ± 1.86%). Further, all *E. faecalis* strains showed good survival (122.32 ± 1.12% to 119.97 ± 0.16%) in the presence of pancreatic enzyme for 4 h. Although the viabilities of *E. faecalis* strains and *L. plantarum* ATCC 14917 in a medium containing bile salts decreased after 4 h, the percentage of survival of every *E. faecalis* strain in bile salts was significantly higher than that of *L. plantarum* ATCC 14917 (75.68 ± 6.43% to 74.16 ± 5.14% vs. 30.97 ± 0.37%).

**Table 1:** Tolerance test under simulated gastrointestinal tract conditions of *E. faecalis* strains.

**Survival %**	**PK1003**	**PK1201**	**PK1202**	**PK1301**	**PK1302**	**PK1801**	**PK2003**	**PK2004**	**PK2502**	***L. plantarum* ATCC14917**
pH = 2	85.48 ± 2.21^*^	88.69 ± 2.67^*^	82.45 ± 4.58^*^	93.78 ± 6.38^*^	91.82 ± 7.06^*^	93.68 ± 1.34^*^	88.11 ± 6.49^*^	96.55 ± 1.52^*^	67.25 ± 2.01^*^	UD
pH = 3	123.19 ± 2.38^*^	122.36 ± 1.18^*^	122.36 ± 1.18^*^	100.37 ± 1.42^*^	120.73 ± 3.17^*^	125.74 ± 1.68^*^	125.23 ± 0.74^*^	124.48 ± 1.35^*^	124.82 ± 1.57^*^	38.63 ± 1.73
pH = 4	124.38 ± 1.40	124.09 ± 3.22^*^	122.79 ± 0.37^*^	126.93 ± 0.11^*^	123.52 ± 2.82^*^	126.06 ± 1.82^*^	126.54 ± 0.99^*^	125.78 ± 1.01^*^	124.83 ± 0.95^*^	102.18 ± 3.26
Pepsin	36.94 ± 1.92^*^	53.18 ± 1.76^*^	43.65 ± 2.73^*^	39.18 ± 1.64^*^	38.08 ± 0.12^*^	49.97 ± 1.47^*^	39.55 ± 1.47^*^	46.55 ± 1.86^*^	54.06 ± 1.72^*^	UD
Pancreatic enzyme	121.89 ± 0.79^*^	119.97 ± 0.16^*^	120.83 ± 1.61^*^	120.21 ± 1.61^*^	120.35 ± 1.59^*^	122.14 ± 0.39^*^	122.32 ± 1.12^*^	121.38 ± 0.76^*^	120.41 ± 3.15^*^	109.87 ± 2.23
Bile salts	74.16 ± 5.14^*^	75.68 ± 6.43^*^	73.40 ± 7.93^*^	67.21 ± 7.99^*^	75.8 ± 8.04^*^	66.77 ± 8.29^*^	68.39 ± 7.36^*^	65.38 ± 6.11^*^	66.22 ± 3.76^*^	30.97 ± 0.37

* *p < 0.05; UD, undetectable*.

**Figure 1:**
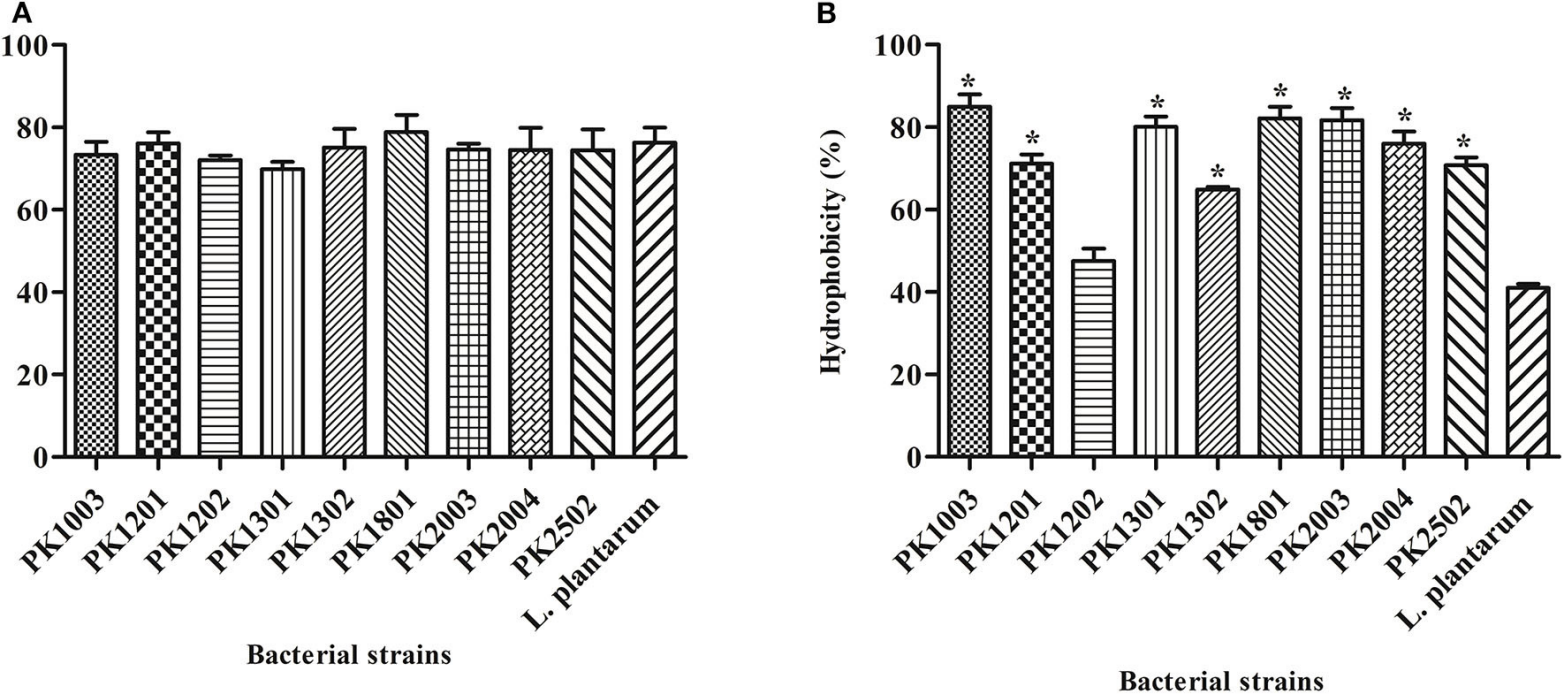
Adhesion of *E. faecalis* isolates and *L. plantarum* ATCC 14917 to a human cell line. **(A)** Adhesion ability. **(B)** % Hydrophobicity. Error bars indicate the standard deviation of three independent experiments. *p < 0.05.

The hydrophobicity of the nine *E. faecalis* strains studied here varied from 47.51 ± 3.02% to 85.00 ± 2.93%. The hydrophobicity of every strain, except *E. faecalis* PK1202, was significantly higher than that of *L. plantarum* ATCC 14917 (41.08 ± 0.89%) (Figure 1A). Human intestinal cell adhesion assay was performed to confirm the adhesion of the nine *E. faecalis* strains to intestinal epithelial HT-29 cells. The results showed that nine *E. faecalis* strains adhered more strongly to HT-29 cells as compared to *L. plantarum* ATCC 14917. *E. faecalis* PK1801 showed the highest adhesiveness (78.83 ± 4.16%) (Figure 1B).

### Safety of the *E. faecalis* Isolates

The antibiotic susceptibility profiles of *E. faecalis* strains are listed in Table 2. All nine strains were susceptible to ampicillin, penicillin, imipenem, and vancomycin, but were resistant to gentamicin (100%). Five *E. faecalis* strains (PK1003, PK1301, PK2003, PK2004, and PK2502) were resistant to erythromycin (55.56%), and tetracycline (66.67%), and only *E. faecalis* PK1801 was resistant to tetracycline. Interestingly, none of the isolated strains were resistant to vancomycin. PCR analysis did not detect *Van-A* or *Van-B* genes. Moreover, the results of PCR screening for the presence of enterococcal virulence genes revealed that the strains harbored different gene patterns (Table 3). The genes *cpd, cob, ccf*, and *cad* encode sex pheromone determinants that facilitate bacterial conjugation. While *cpd* and *cob* were carried by some *E. faecalis* strains, *ccf* and *cad* were not detected in any of the *E. faecalis* strains. All nine strains carried *efaAfs* and *gel*E genes, which are involved in cell adhesion and encoding of gelatinase, respectively.

**Table 2:** Antibiotic susceptibility of *Enterococcus* strains.

**Strains**	**Ampicillin (10 μg)**	**Penicillin (10 μg)**	**Imipenem (10 μg)**	**Vancomycin (30 μg)**	**Gentamicin (10 μg)**	**Erythromycin (15 μg)**	**Tetracycline (30 μg)**	**Ciprofloxacin (5 μg)**
PK1003	S	S	S	S	R	R	R	S
PK1201	S	S	S	S	R	I	S	I
PK1202	S	S	S	S	R	I	S	I
PK1301	S	S	S	S	R	R	R	S
PK1302	S	S	S	S	R	I	S	S
PK1801	S	S	S	S	R	I	R	I
PK2003	S	S	S	S	R	R	R	I
PK2004	S	S	S	S	R	R	R	I
PK2502	S	S	S	S	R	R	R	I
Resistant rate (%)	0 (0/9)	0 (0/9)	0 (0/9)	0 (0/9)	100 (9/9)	55.56 (5/9)	66.67 (6/9)	0 (0/9)

*R, resistant; I, intermediate; S, susceptible*.

**Table 3:** Summary of polymerase chain reaction assays and phenotypic screening for virulence determinants.

**Strains**	**Genotype**	**Phenotype**
PK1003	*Agg*^+^, *gel*E^+^, *cpd*^+^, *efaA_*fs*_*^+^	None
PK1201	*gel*E^+^, *cpd*^+^, *efaA_*fs*_*^+^, *cob*^+^	None
PK1202	*Agg*^+^, *gel*E^+^, *cpd*^+^, *efaA_*fs*_*^+^, *cob*^+^	None
PK1301	*Agg*^+^, *gel*E^+^, *cpd*^+^, *efaA_*fs*_*^+^	None
PK1302	*gel*E^+^, *cpd*^+^, *efaA_*fs*_*^+^	None
PK1801	*gel*E^+^, esp^+^, *cpd*^+^, *efaA_*fs*_*^+^	None
PK2003	*Agg*^+^, *gel*E^+^, *cyl*M^+^, *cyl*B^+^, *cyl*A^+^, *esp*^+^, *cpd*^+^, *efaA_*fs*_*^+^, *cob*^+^	Hemolytic activity^**+**^
PK2004	*Agg*^+^, *gel*E^+^, *cyl*M^+^, *cyl*B^+^, *cyl*A^+^, *esp*^+^, *cpd*^+^, *efaA_*fs*_*^+^, *cob*^+^	Hemolytic activity^**+**^
PK2502	*gel*E^+^, *cyl*M^+^, *cyl*B^+^, *cyl*A^+^, *esp*^+^, *cpd*^+^, *efaA_*fs*_*^+^, *cob*^+^	Hemolytic activity^**+**^

+, *Positive*.

Phenotypic assays demonstrated that none of the nine *E. faecalis* strains had detectable gelatinase activity or were able to degrade mucin. Moreover, three *E. faecalis* strains that carried *esp* showed hemolytic activity on blood agar plates. Therefore, these strains were excluded as potential probiotics.

The virulence of the remaining six strains were judged to be safe and confirmed using the *G. mellonella* killing assay. The *E. faecalis* ATCC 4736, a pathogenic strain, could kill *G. mellonella* larvae (85%). In contrast, the survival rates of the larvae ranged from 80 to 100% when they were injected with the six *E. faecalis* strains, even at high doses of *E. faecalis*. Similar results were observed with *L. plantarum* ATCC 14917. These data suggested that these six *E. faecalis* strains were safe for use as potential probiotics.

### Bacteriocin Mediated Antimicrobial Activity of *E. faecalis*

Primarily, the six *E. faecalis* isolates exerted their antimicrobial activity against *C. difficile*. Zones of inhibition were in the range of 10.90 ± 0.10 to 14.00 ± 0.00 mm. To ascertain the involvement of proteinaceous agents, the cell-free supernatants of *E. faecalis* strains were neutralized to pH 7.2 and treated with proteinase K to digest soluble proteins within the supernatant. A clear zone of inhibition was undetectable, indicating that the inhibitory activity of *E. faecalis* strains against toxigenic *C. difficile* was mediated by bacteriocins. In addition, while BSH activity was detected in all *E. faecalis* strains except PK1301 and PK1302, production of H_2_O_2_ was not detected in any of the six selected *E. faecalis* strains.

### Potential Probiotic *E. faecalis* Activity Against Toxigenic *C. difficile* Strains and Their Spore Production and Germination

An essential property of a probiotic is its ability to inhibit the growth of bacterial pathogens. The selected six *E. faecalis* strains showed strong ability to inhibit four toxigenic *C. difficile* strains including *C. difficile* ATCC 630, *C. difficile* ATCC 43255, and two clinical isolates (*C. difficile* 17 and *C. difficile* 541) (Table 4). To establish the probiotic nature of the six *E. faecalis* strains, we studied their ability to inhibit sporulation and spore production in toxigenic *C. difficile* strains.

**Table 4:** Inhibition of toxigenic *C. difficile* strains by *Enterococcus* isolates (mm).

**Pathogenic bacteria**	**PK1003**	**PK1201**	**PK1202**	**PK1301**	**PK1302**	**PK1801**
*C. difficile* ATCC 630	13.30 ± 0.20	-	-	12.90 ± 0.36	-	14.00 ± 0.00
*C. difficile* ATCC 43255	11.27 ± 0.06	11.43 ± 0.51	11.00 ± 0.10	10.90 ± 0.10	11.20 ± 0.30	11.00 ± 0.10
Clinical *C. difficile* 17	11.33 ± 1.15	12.00 ± 1.00	12.00 ± 0.00	12.67 ± 0.58	12.33 ± 2.08	12.67 ± 2.31
Clinical *C. difficile* 541	13.00 ± 1.00	12.67 ± 0.58	12.33 ± 0.58	13.33 ± 1.15	13.67 ± 3.21	13.33 ± 0.58

*-, Not inhibit*.

**Figure 2:**
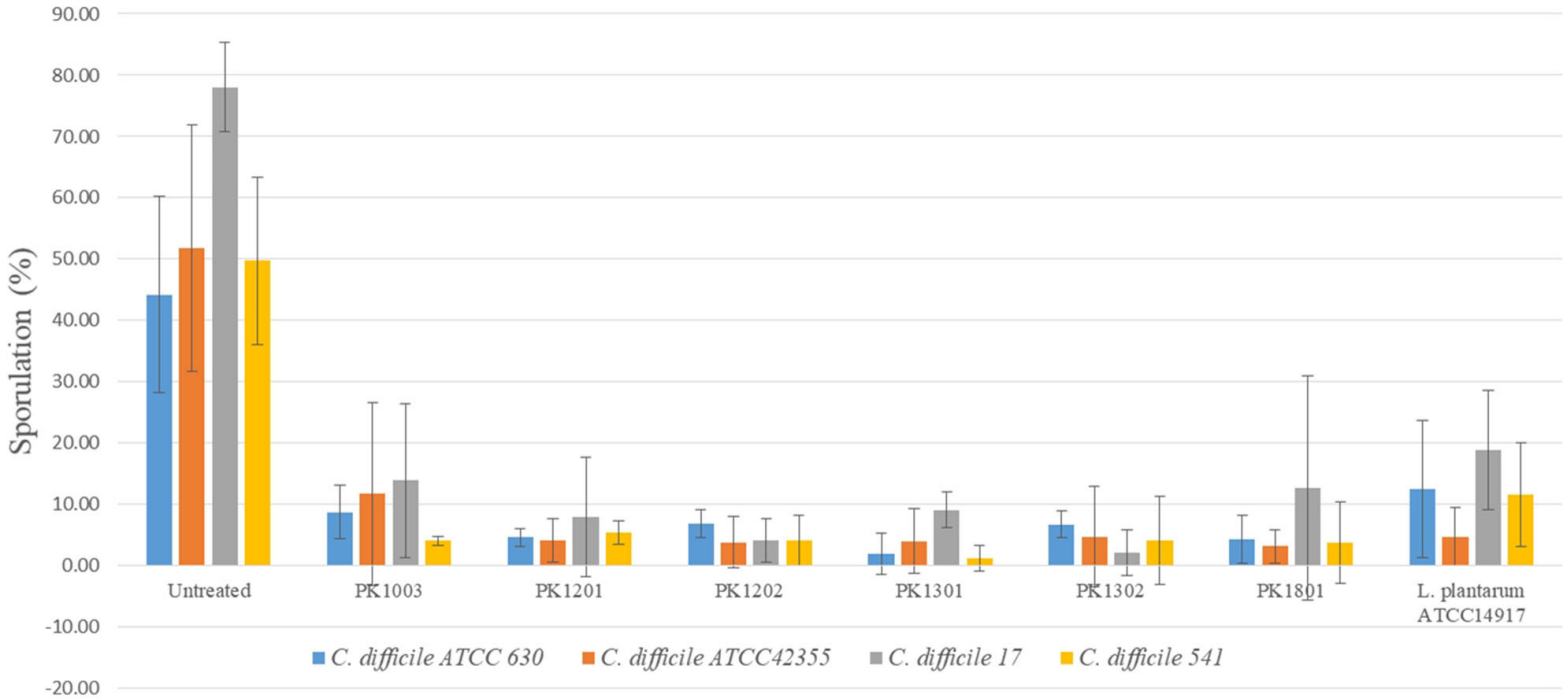
Percentage of *C. difficile* spore with or without treatment with six *E. faecalis* strains and *L. plantarum* ATCC 14917.

The ability to inhibit sporulation was tested for the six *E. faecalis* strains and *L. plantarum* ATCC 14917. The percentage of spore production of the *C. difficile* strains ranged from 44.20 ± 15.97% to 78.07 ± 7.30%. Following treatment with the six *E. faecalis* strains and *L. plantarum* ATCC 14917, the percentage of sporulation of *C. difficile* reduced (1.19 ± 2.06% to 13.89 ± 12.49%) significantly compared to that of untreated ones (Figure 2).

The six selected *E. faecalis* strains and *L. plantarum* ATCC 14917 were screened for their potential probiotics activity against *C. difficile* by examining their ability to inhibit spore germination. The results showed that exposure of the spores of toxigenic *C. difficile* strains to the *E. faecalis* strains and *L. plantarum* ATCC 14917 led to an utter reduction in their germination (0%−1.80 ± 2.17%) (Table 5), indicating probiotic properties for all the six *E. faecalis* strains.

**Table 5:** Relative percentage of germination of toxigenic *C difficile* when treated with *E. faecalis* isolates.

**Strains**	***C. difficile* ATCC 630**	***C. difficile* ATCC 43255**	***C. difficile* 17**	***C. difficile* 541**
*E. faecalis* PK1003	0.01 ± 0.02	0.01 ± 0.02	0.13 ± 0.13	0.17 ± 0.24
*E. faecalis* PK1201	0.01 ± 0.01	0.01 ± 0.02	0.05 ± 0.05	1.67 ± 2.36
*E. faecalis* PK1202	0.01 ± 0.02	0.01 ± 0.02	0.13 ± 0.13	0.17 ± 0.24
*E. faecalis* PK1301	0.00 ± 0.00	0.01 ± 0.02	0.00 ± 0.00	0.32 ± 0.02
*E. faecalis* PK1302	0.00 ± 0.00	0.00 ± 0.01	0.00 ± 0.00	0.01 ± 0.01
*E. faecalis* PK1801	0.01 ± 0.02	0.13 ± 0.18	0.14 ± 0.14	1.80 ± 2.17
*L. plantarum* ATCC14917	0.00 ± 0.00	0.01 ± 0.02	0.03 ± 0.03	0.05 ± 0.02

### Effect of Co-culture of Isolated *E. faecalis* and Toxigenic *C. difficile* on HT-29 Cells

**Figure 3:**
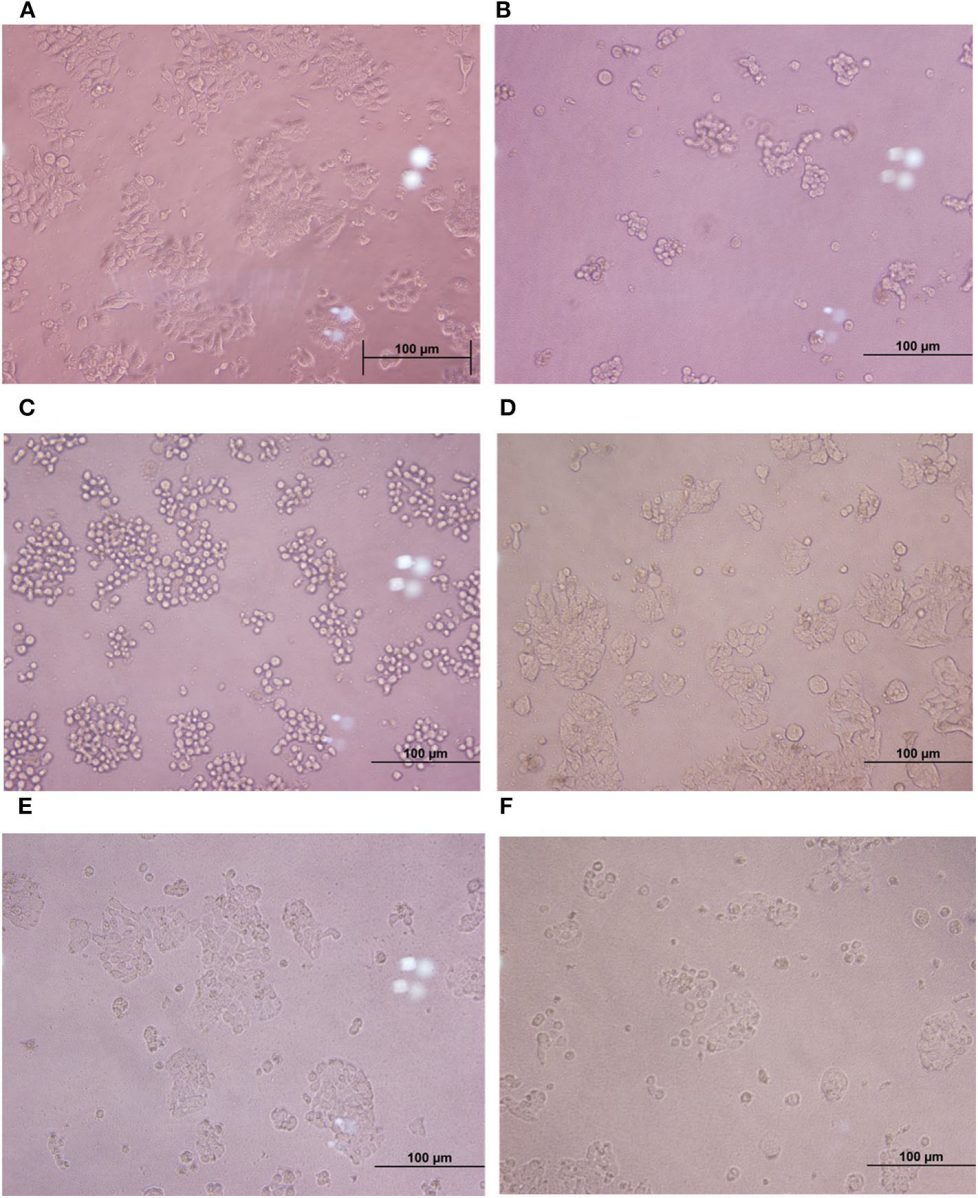
Comparison of cytotoxic effects of individual *C. difficile* strains, *E. faecalis*, and co-culture of *E. faecalis* with *C. difficile* on HT-29 cells. **(A)** HT-29 monolayer cells. **(B–D)** HT-29 cells treated with the cell-free supernatant from toxigenic *C. difficile* strain and *E. faecalis*. **(E, F)** HT-29 cells treated with the cell-free supernatant from co-culture of *E. faecalis* and *C. difficile*.

Cytopathic effects of toxigenic *C. difficile* on HT-29 cells were evaluated by cytotoxicity assay. The results showed that the treatment of HT-29 cells with the cell-free supernatant of the four *C. difficile* strains (ATCC 630, ATCC 43255, and clinical strains 17 and 541) led to changes in the morphology of the cells (more than 50% of the cells were round and became spherical), whereas the treatment with the cell-free supernatant of six monoculture *E. faecalis* strains and monoculture *L. plantarum* ATCC 14917 caused only 5–10% of the HT-29 cells to exhibit rounded morphology, similar to negative control (Figure 3). Moreover, when HT-29 cells were treated with the cell-free *E. faecalis* supernatants that were previously incubated with clinical *C. difficile* 541, they remained stable and the percentage of cell rounding was decreased when compared with HT-29 cells treated with the cell-free supernatant of the four *C. difficile* strains. However, when HT-29 cells were treated with a co-culture of the cell-free supernatant from six *E. faecalis* incubated with *C. difficile* ATCC 630, ATCC 43255, and clinical *C. difficile* 17, >50% were rounded. Nevertheless, these cell injuries were less than that observed following treatment with the cell-free supernatant of *C. difficile* monoculture (Figure 3).

**Figure 4:**
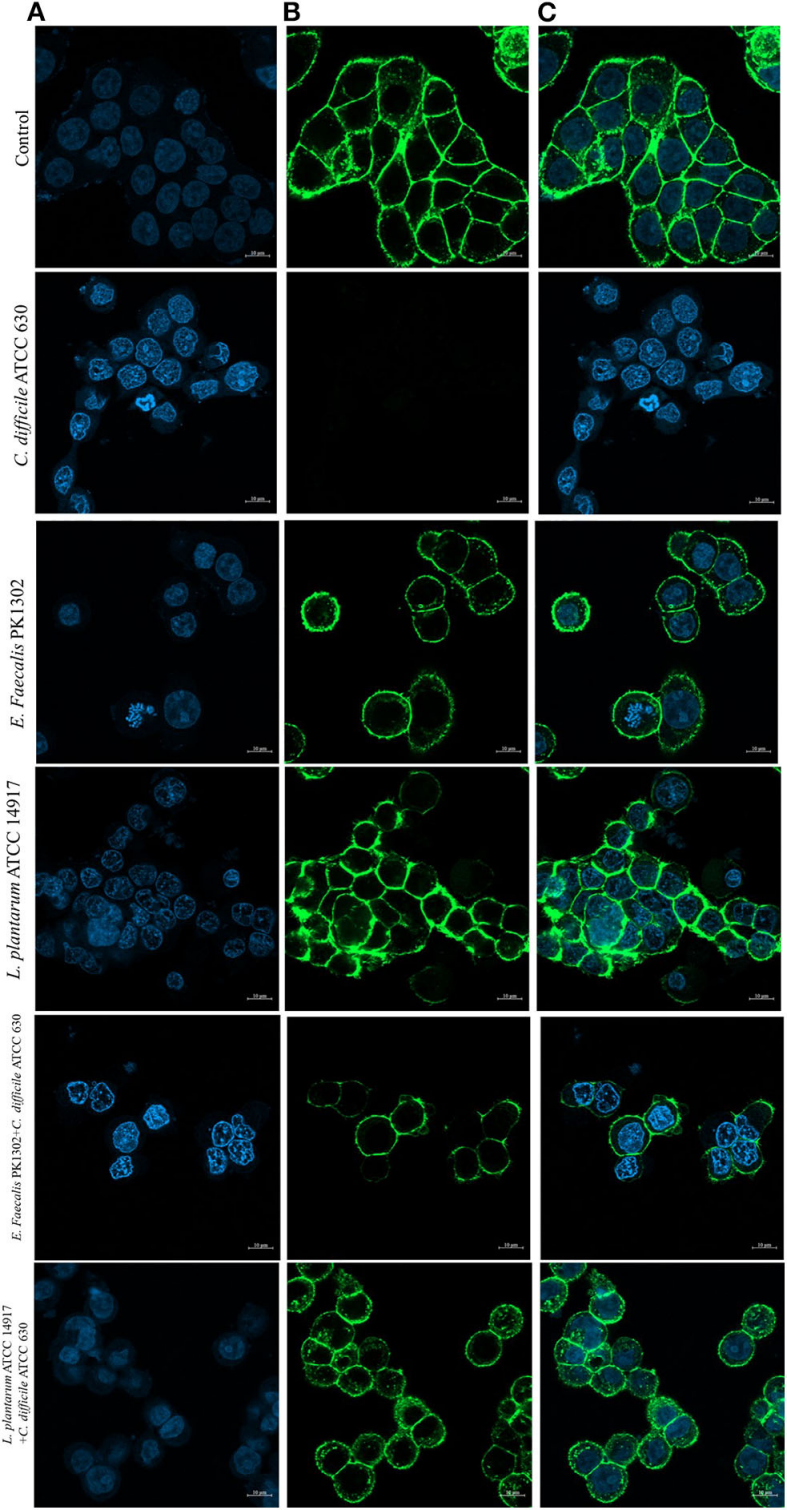
Immunofluorescence images obtained by confocal scanning laser microscopy of HT-29 cells after 24 h with the cell-free supernatant of individual *C. difficile* ATCC630, *E. faecalis* 1302, and *L. plantarum* ATCC 14917 and with the cell-free supernatant obtained after incubation of *E. faecalis* with *C. difficile* ATCC630 and *L. plantarum* ATCC 14917 with *C. difficile* ATCC630. **(A)** DAPI-stained nucleus (blue, excited at 405 nm). **(B)** F-actin stained with Phalloidin-Alexa-Fluor-488 probe (green, excited at 490 nm). **(C)** Combination of nucleus and F-actin stained.

Furthermore, to study the cellular events triggered by different treatments, *F*-actin detection was performed using an immunofluorescence assay. The results showed that while HT-29 cells of the control group exhibited a typical *F*-actin cytoskeleton, imbibed nucleus, and connected cells (Figure 4), the HT-29 cells treated with the cell-free supernatant of individual of *C. difficile* monoculture showed loss of *F*-actin cytoskeleton-mediated interconnections between cells and also exhibited condensed nuclei, indicating the initial stage of apoptosis. HT-29 cells became rounded and tight junctions were disrupted. The images of HT-29 cells monocultured with the cell-free supernatant of six *E. faecalis* or *L. plantarum* ATCC 14917 were similar to that of the control group and *F-*actin showed normal morphology. The intensity of DAPI staining of the nucleus of these cells was comparable to that of control, but less than the HT-29 cells that were treated with the cell-free supernatant of individual *C. difficile*.

The confocal images of HT-29 cells treated with cell-free supernatants obtained from the co-culture of *E. faecalis* PK1302 or *L. plantarum* ATCC 14917 with each of the four toxigenic *C. difficile* showed that HT-29 cells were less damaged when compared with HT-29 cells that were treated with the cell-free supernatant of individual monoculture *C. difficile*. Some parts of *F*-actin cytoskeleton showed interconnected structure, and the cells exhibited normal morphology. While the nuclei of most cells were similar to those in the control group, some nuclei were condensed.

Overall, these results suggested that the cell-free supernatants of *E. faecalis* reduced the cytopathic effects of *C. difficile* by counteracting the effect of *C. difficile* toxins.

## Discussion

Probiotics are being explored as an alternative therapeutic option for treatment and prevention of CDI (9). The choice of the appropriate probiotic against CDI is highly relevant. This is mainly because not all probiotic formulations are effective against CDI (35, 36).

Various strains of potential probiotics have been isolated from human and animal feces, particularly because this source is generally recognized as safe for human consumption (16, 37). Enterococci, specifically *E. faecium* and *E. faecalis*, are predominantly present in the normal flora of the intestinal tract of warm-blooded animals. They confer health benefits to their host (17). Therefore, new strains of enterococci from the feces are typically screened for potential probiotic properties. The strains isolated from the feces of breast-fed infant possess higher ability to survive the passage through the GIT condition compared to the strains isolated from dairy food. They compete with pathogenic bacteria for nutrients and colonize GIT effectively (38). Moreover, several studies have reported new probiotics from feces of breast-fed infant since they are dominated by bifidobacteria, lactobacilli, enterococci, and other LABs (23, 39–41). In this study, nine *E. faecalis* strains, PK1003, PK1201, PK1202, PK1301, PK1302, PK180, PK2003, PK2004, and PK2502, were isolated from the feces of breast-fed infants for their potent tolerance toward GIT conditions. The ability to attach to the intestinal mucosa is an important property of a potential probiotic. The adhesion of probiotic bacteria to human epithelial cells may serve as an important mechanism for preventing the pathogens from colonization and for preventing the removal of bacteria from the colon through peristalsis (42, 43). Adhesion to GIT is mainly associated with the hydrophobicity of the bacterial cell surface (44). Here, all *E. faecalis* strains showed high hydrophobicity, suggesting that they were able to adhere to intestinal epithelial cells.

*E. faecalis* is commonly used as a food product and as a dairy starter culture. They were recently used as probiotics for therapeutic treatments, and no adverse effects have been reported so far (16). However, some *E. faecalis* strains are nosocomial pathogens. Therefore, unless a probiotic is declared as “generally recognized as safe,” their safety parameters must be determined before use. Antibiotics resistance is a critical factor that needs to be evaluated to assess the safety of enterococci, and it needs to be ensured that they do not harbor acquired and transferable determinants of antibiotic resistance. In particular, vancomycin resistance of enterococci is a major safety concern for probiotics, because it is horizontally transferred to other strains (45). Interestingly, all isolated strains were sensitive to vancomycin and did not harbor the vancomycin-resistant genes *Van-A* and *Van-B*. All strains carried at least one virulent determinant gene; however, the presence of virulence genes does not indicate pathogenic property. Of the nine strains, six *E. faecalis* strains presented *efaAfs* and *gel*E; *efaAfs* are involved in cell adhesion of bacterial pathogens (28, 46), which, in turn, is important for probiotics to adhere to intestinal cells. *gel*E encodes gelatinase that hydrolyzes collagen, casein, and hemoglobin (28, 46). Three *E. faecalis* strains (PK2003, PK2004, and PK2502) that carried *esp* showed hemolytic activity on blood agar plates. Therefore, these strains were excluded as potential probiotics. The virulence of the six strains was also determined using the *G. mellonella* killing assay that is a useful model for studying infections of human pathogens, because the innate immune system of the *G. mellonella* larvae is similar to that of vertebrates (47). The six selected *E. faecalis* strains did not kill *G. mellonella*. Taken together, these results support the fact that all six *E. faecalis* strains could be used as potential probiotic strains, which were safe and which met all requirements of probiotic properties.

The probiotics are known to act against CDI through different bacterial antagonistic mechanisms, such as competition for adhesion to intestinal mucosa, producing antimicrobial molecules, modulation of intestinal inflammation, reduction of toxicity caused by *C. difficile*, and inhibition of *C. difficile* spores (48). Some compounds produced by probiotic bacteria, such as metabolites, organic acids, and bacteriocins, may also contribute to the antimicrobial activity against enteropathogens (49). *Enterococcus* spp. can produce enterocins, such as Enterocin A, Enterocin AS-48, and Enterocins L50A and L50B that can form pores in the cell membrane, deplete the transmembrane potential, and/or the pH gradient leading to the leakage of indispensable intracellular molecules and cell lysis (14, 16, 50). In this study, bacteriocin was detected in all of the six selected *E. faecalis* strains that can inhibit toxigenic *C. difficile*.

*C. difficile* spores are important for disease transmission. They are resistant to numerous environmental stresses, including low pH, heat, radiation, and chemicals (51). Furthermore, failure to eliminate *C. difficile* spores can lead to recurrence of CDI within 2–3 months (52). Currently, probiotics have been used in some hospitals for reducing *C. difficile* spores in patients who are administered with antibiotics (53). The results of the study by Rätsep et al. (15) showed that a combination of xylitol with *L. plantarum inducia* suppresses the germination of spores and outgrowth into vegetative toxin-producing cells of *C. difficile* and also reduces the gut colonization of the pathogen, which subsequently reduced the CDI rates. In terms of inhibition, the six *E. faecalis* strains identified in this study also exhibited significant inhibitory effects on spore production and germination of *C. difficile*. Almost all the *E. faecalis* strains isolated in this study produced BSH enzymes, which is implicated in the inhibition of spore germination of *C. difficile* (10, 54).

*C. difficile* produce toxins, mainly enterotoxin (TcdA) and/or cytotoxin (TcdB). These toxins disrupt the actin cytoskeleton and tight junctions leading to disorganize the F-actin cytoskeleton and tight junctions of intestinal epithelial cells, morphological changes, and subsequent cell death (4, 5). In the previous study, enterococci were shown to be effective in the prevention of AAD (16). *E. faecalis* NM815, *E. faecalis* NM915, and *E. faecium* NM1015 were shown to inhibit *C. difficile* (12). Similarly, our results showed that six *E. faecalis* strains were able to inhibit *C. difficile* by producing bacteriocin and/or BSH. In the previous report, some probiotics were found to inhibit *C. difficile* toxins by producing protease proteins that hydrolyzed *TcdA* and *TcdB* and inhibited their binding to the respective intestinal brush border receptors (10, 54). Valdes-Varela et al. screened bifidobacteria and lactobacilli that were able to antagonize the cytotoxic effect of *C. difficile* on the intestinal epithelial HT29 monolayer (34). They showed that *Bifidobacterium longum* and *Bifidobacterium breve* were able to reduce the toxic effects of the pathogen on HT-29 cell lines and rounding was prevented and *F*-actin microstructure and tight junctions between adjacent cells were preserved. *E. faecium* and *L. lactis* have also been shown to help protect epithelial cells from *C. difficile* toxins (11). Similarly, the results of our study showed that six *E. faecalis* may secrete antibacterial agents that reduce the cytotoxic effects of toxins of *C. difficile* and protect HT-29 cells.

## Conclusion

Six *E. faecalis* strains were identified as potential probiotics for preventing or controlling *C. difficile* colonization or CDI. They were found to inhibit toxigenic *C. difficile* by reducing the clostridial toxic effects on HT-29 cells and preventing *C. difficile* spore production and germination. However, further *in vivo* studies into the inhibition of *C. difficile* using these *E. faecalis* strains are required.

## Data Availability Statement

The original contributions presented in the study are included in the article/Supplementary Material, further inquiries can be directed to the corresponding author/s.

## Ethics Statement

The studies involving human participants were reviewed and approved by The Ethics Committees of the Faculty of Medicine, Prince of Songkla University. Written informed consent to participate in this study was provided by the participants' legal guardian/next of kin.

## Author Contributions

CR, AT, and KS conceived and designed the experiments. CR, NI, PS, and WC performed the experiments. CR and KS analyzed the data. KS contributed reagents, materials, and analysis tools. CR, AA, and KS wrote the manuscript. All authors contributed to the article and approved the submitted version.

## Conflict of Interest

The authors declare that the research was conducted in the absence of any commercial or financial relationships that could be construed as a potential conflict of interest.
