# Supplementary material for: Enterococcus faecalis Isolated From Infant Feces Inhibits Toxigenic Clostridioides (Clostridium) difficile
Source: Front Pediatr. 2020 Sep 25;8:572633. doi: 10.3389/fped.2020.572633 (PMC7545477; doi:10.3389/fped.2020.572633)
Supplement: Supplementary file 1 [file Table_1.docx]

**Supplementary Table 1.** PCR primers for amplifying sequences encoding enterococcal virulence factors and predicted amplicon sizes

| Gene | Primers | Sequence (5’🡪3’) | Expected product (bp) | Ref. |
| --- | --- | --- | --- | --- |
| *Agg_2_* | TE32 | GTTGTTTTAGCAATGGGGTAT | 1210 | Reviriego C., et al. (2005) |
|  | TE33 | CACTACTTGTAAATTCATAGA |  |  |
| *gelE* | TE9 | ACCCCGTATCATTGGTTT | 419 |  |
|  | TE10 | ACGCATTGCTTTTCCATC |  |  |
| *cylM* | TE13 | CTGATGGAAAGAAGATAGTAT | 742 |  |
|  | TE14 | TGAGTTGGTCTGATTACATTT |  |  |
| *cylB* | TE15 | ATTCCTACCTATGTTCTGTTA | 843 |  |
|  | TE16 | AATAAACTCTTCTTTTCCAAC |  |  |
| *cylA* | TE17 | TGGATGATAGTGATAGGAAGT | 517 |  |
|  | TE18 | TCTACAGTAAATCTTTCGTCA |  |  |
| *esp* | TE34 | TTGCTAATGCTAGTCCACGACC | 933 |  |
|  | TE36 | GCGTCAACACTTGCATTGCCGAA |  |  |
| *cpd* | TE51 | TGGTGGGTTATTTTTCAATCC | 782 |  |
|  | TE52 | TACGGCTCTGGCTTACTA |  |  |
| *espfs* | TE34 | TTGCTAATGCTAGTCCACGACC | 933 |  |
|  | TE36 | GCGTCAACACTTGCATTGCCGAA |  |  |
| *cob* | TE49 | AACATTCAGCAAACAAAGC | 1405 |  |
|  | TE50 | TTGTCATAAAGAGTGGTCAT |  |  |
| *ccf* | TE53 | GGGAATTGAGTAGTGAAGAAG | 543 |  |
|  | TE54 | AGCCGCTAAAATCGGTAAAAT |  |  |
| *cad* | E42a | TTGCTTTGTCATTGACAATCCG | 1299 |  |
|  | TE43a | ACTTTTTCCCAACCCCTCAA |  |  |
| *vanA* | vanA-F | GGGAAAACGACAATTGC | 732 | Praharaj I., et al. (2013) |
|  | vanA-R | GTACAATGCGGCCGTTA |  |  |
| *vanB* | vanB-F | ACGGAATGGGAAGCCGA | 647 |  |
|  | vanB-R | TGCACCCGATTTCGTTC |  |  |
